# Molecular patterns of isolated tubulitis differ from tubulitis with interstitial inflammation in early indication biopsies of kidney allografts

**DOI:** 10.1038/s41598-020-79332-9

**Published:** 2020-12-17

**Authors:** Petra Hruba, Katelynn Madill-Thomsen, Martina Mackova, Jiri Klema, Jana Maluskova, Ludek Voska, Alena Parikova, Janka Slatinska, Philip F. Halloran, Ondrej Viklicky

**Affiliations:** 1 Transplant Laboratory, Institute for Clinical and Experimental Medicine, 140 21 Prague, Czech Republic; 2 Alberta Transplant Applied Genomics Centre, Edmonton, AB T6G 2S2 Canada; 3 University of Alberta, Edmonton, AB T6G 2S2 Canada; 4 Department of Computer Science, Faculty of Electrical Engineering, Czech Technical University, 121 35 Prague, Czech Republic; 5 Department of Pathology, Institute for Clinical and Experimental Medicine, 140 21 Prague, Czech Republic; 6 Department of Nephrology, Transplant Centre, Institute for Clinical and Experimental Medicine, Videnska 1958/9, 14021 Prague, Czech Republic

**Keywords:** Molecular medicine, Nephrology

## Abstract

The Banff 2019 kidney allograft pathology update excluded isolated tubulitis without interstitial inflammation (ISO-T) from the category of borderline (suspicious) for acute T cell-mediated rejection due to its proposed benign clinical outcome. In this study, we explored the molecular assessment of ISO-T. ISO-T or interstitial inflammation with tubulitis (I + T) was diagnosed in indication biopsies within the first 14 postoperative days. The molecular phenotype of ISO-T was compared to I + T either by using RNA sequencing (n = 16) or by Molecular Microscope Diagnostic System (MMDx, n = 51). RNA sequencing showed lower expression of genes related to interferon-y (p = 1.5 *10^–16^), cytokine signaling (p = 2.1 *10^–20^) and inflammatory response (p = 1.0*10^–13^) in the ISO-T group than in I + T group. Transcripts with increased expression in the I + T group overlapped significantly with previously described pathogenesis-based transcript sets associated with cytotoxic and effector T cell transcripts, and with T cell-mediated rejection (TCMR). MMDx classified 25/32 (78%) ISO-T biopsies and 12/19 (63%) I + T biopsies as no-rejection. ISO-T had significantly lower MMDx scores for interstitial inflammation (p = 0.014), tubulitis (p = 0.035) and TCMR (p = 0.016) compared to I + T. Fewer molecular signals of inflammation in isolated tubulitis suggest that this is also a benign phenotype on a molecular level.

## Introduction

In kidney transplantation, the Banff classification of renal allograft pathology is used to guide therapy based on interpretation of individual histological lesions scores. It is focused primarily on the diagnosis of rejection (either T-cell mediated (TCMR), antibody–mediated or mixed rejection). The Banff category of borderline (suspicious) for acute TCMR (BL) describes changes insufficient for a diagnosis of rejection, the clinical significance of which has been widely debated in recent years^1–3^. BL was introduced into the Banff classification as early as 1997^4^, and besides tubulitis this category required inflammation in at least 10% of non-scarred cortex tissue (Banff i > 0). Between 2005–2019, the Banff definition^5^ of BL also involved isolated tubulitis without inflammation. The most recent update of the Banff classification^6^ eliminated isolated tubulitis due to its benign outcome, based on the Nankivell et al. study which reported that the 5-year graft survival of isolated tubulitis (ISO-T) was similar to normal biopsies (i0t0)^7^.

Molecular assessment of allograft tissue offers an innovative tool to improve both clinical diagnostics and our understanding of the biological processes underlying particular graft pathologies^8–12^. Interestingly, studies which used the Molecular Microscope Diagnostic System (MMDx) platform identified only 30% of BL biopsies (according to previous definitions of BL) as rejection, while the majority of findings reflected injury/repair molecular processes^8–10^. Our previous microarray study showed higher immune activation in BL diagnosed in indication biopsies early after transplantation compared to later biopsies^12^ and such immune activation may represent a long-term risk. In all these studies, the definition of BL included isolated tubulitis. Little is known about whether ISO-T shares some similarities in terms of biology and outcomes with inflammation and tubulitis (I + T).

In this study, we used two different molecular techniques, RNA sequencing and MMDx, in early indication biopsies diagnosed as ISO-T and I + T by histology and found that those categories clearly differed on molecular level.

## Results

### Molecular phenotype of ISO-T and I + T assessed by RNAseq

Differential gene expression analysis between ISO-T and I + T categories was performed using RNA sequencing to reveal the unique biological processes. RNA sequencing was carried out in a cohort of early indication biopsies, median 8 days post-transplantation (range 5–18) diagnosed as either ISO-T (n = 8) or I + T (n = 8). Groups did not differ in terms of transplant demographic and outcome (Table S1, Figure S1).

**Figure 1:**
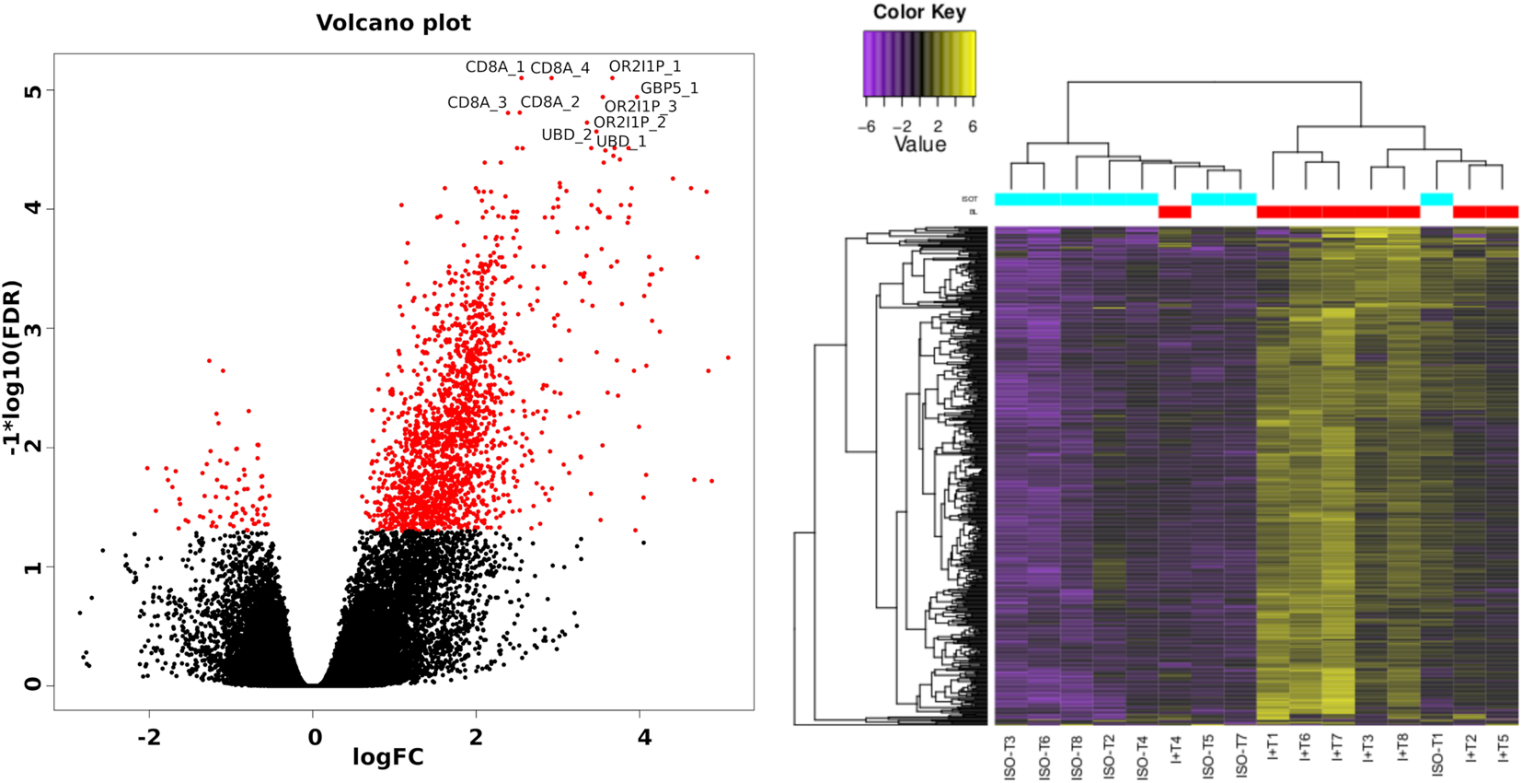
Volcano plots and heatmaps of differentially expressed transcripts between ISO-T and I + T.

461 transcripts coding for 157 unique genes were increased in expression, and only 1 transcript decreased in expression in I + T group compared to ISO-T group (fold change > 4, adjusted p value < 0.05, Fig. 1). Heatmaps of differentially expressed transcripts in ISO-T group formed a homogenous cohort in all but one sample (Fig. 1). Interestingly, the only patient from the ISO-T group who displayed molecular signals typical of the I + T category had undergone a biopsy diagnosed as Banff TCMR IB at one year post-transplant and another diagnosed as Banff chronic antibody-mediated rejection at 3 years.

Gene annotation analysis of increased transcripts in I + T vs. ISO-T showed activation of a cytokine-mediated signalling pathway (p = 2.1*10^–20^), cellular response to interferon-gamma (p = 1.5 *10^–16^), inflammatory response (p = 1.0 *10^–13^) and other GO terms associated with lymphocyte chemotaxis, cytokine production and T cell activation (Table 1). From the list of differentially expressed transcripts, 18 transcripts were able to discriminate between ISO-T and I + T (AUC = 1 and p < 000.1, Table 2). These transcripts corresponded to only 7 unique genes: *OR2I1P* (olfactory receptor family 2 pseudogene), *GBP1* and *GBP5* (guanylate binding protein 1 and 5), *UBD* (ubiquitin D), *IDO1 (*indoleamine 2,3-dioxygenase 1)*, CXCR2P1* (C-X-C motif chemokine receptor 2 pseudogene 1) and chemokine *CXCL10*. The G-protein-coupled receptor *OR2I1P* was also among the top 30 transcripts for the MMDx TCMR classifier. MMDx annotated *GBP1*, *GBP5*, and *UBD* as induced by IFNy and rejection^13^.

**Table 1:** Gene annotation analysis of transcripts increased in I + T compared to ISO-T (461 transcripts with FC > 4, adjusted p value < 0.05).

	GO term	Adjusted P-value	Genes
GO:0019221	Cytokine-mediated signaling pathway	2.10E−20	*CXCL9;CD80;EBI3;ITGB2;CSF2RB;CXCL13;IL2RG;TNF;TNFSF13B;CCL4;IL21R;CCL3; CCL19;CCR5;IL12RB1;HLA-DQA2;GBP1;CCL17;HLA-DPA1;HLA-B;* *HLA-F;MMP9;PSMB9;ISG20;CXCL10;CXCL11;CD4;AIM2;IFNG;OAS2;IRF1;IL1B;XCL2;* *HLA-DPB1;HLA-DRA;XCL1;LTB;LCP1;IL7R*
GO:0071346	Cellular response to γ-gamma	1.51E−16	*HLA-B;ACOD1;HLA-F;IFNG;OAS2;IRF1;CCL4;XCL2;CCL3;HLA-DPB1;* *HLA-DRA;XCL1;CCL19;IL12RB1;HLA-DQA2;TDGF1;GBP1;CCL17;HLA-DPA1*
GO:0006954	Inflammatory response	1.02E−13	*CXCL9;PLA2G2D;ITGB2;CYBB;FPR2;AOAH;CXCL13;LYZ;ITGAL;TNF;CXCL10;CXCL11; CD6;IL1B; CCL4;XCL2;CCL3;XCL1;TLR8; CCL19;CCL17;APOL3*
GO:0050776	Regulation of immune response	1.26E−11	*ZNF683;TRAC;SH2D1A;ITGB2;HLA-B;IGLV3-1;LILRB1;LILRA1;LILRB2;CXCL13;* *HLA-F;CD3E;ITGAL;CD1B;CD3D;SELL;CD8A;IRF1;SLAMF7;HCST*
GO:0050671	Positive regulation of lymphocyte proliferation	3.74E−10	*PTPRC;CD6;CD80;FCRL3;IL1B;HLA-DPB1;CD38;CCL19;LILRB2;CD3E;TNFSF13B;* *HLA-DPA1*
GO:0042102	Positive regulation of T cell proliferation	1.29E−09	*PTPRC;CD6;CD80;IL1B;EBI3;HLA-DPB1;CCL19;LILRB2;CD3E;IL12RB1;HLA-DPA1*
GO:0042129	Regulation of T cell proliferation	1.35E−09	*SPN;PTPRC;CD6;CD80;IL1B;HLA-DPB1;LILRB1;CCL19;LILRB2;CD3E;HLA-DPA1*
GO:0048247	Lymphocyte chemotaxis	1.43E−09	*CXCL10;CXCL9;CXCL11;CCL4;CCL3;XCL2;XCL1;CCL19;CXCL13;CCL17*
GO:0034341	Response to interferon-γ	3.42E−09	*UBD;CCL4;XCL2;CCL3;ACOD1;XCL1;CCL19;IL12RB1;CCL17;TDGF1;HLA-DPA1*
GO:0070098	Chemokine-mediated signaling pathway	4.96E−09	*CXCL10;CXCL9;CXCL11;CCL4;XCL2;CCL3;XCL1;CCL19;CXCL13;CCL17*
GO:0071345	Cellular response to cytokine stimulus	3.62E−08	*CD80;ITGB2;ACOD1;CSF2RB;IL2RG;TNF;MMP9;CXCL10;CD4;IRF1;IL1B;CCL4;* *XCL2;CCL3;XCL1; CCL19;CCR5;IL12RB1;TDGF1;CCL17;HLA-DPA1*
GO:0050851	Antigen receptor-mediated signaling pathway	8.47E−08	*TRAC;CD3E;CD3D;PSMB9;CD79A;CD4;PTPRC;TRBC2;IGHD; HLA-DPB1;* *HLA-DRA;CTLA4;CD38;LCP2;HLA-DQA2;HLA-DPA1*
GO:0060333	Interferon-γ-mediated signaling pathway	8.67E−08	*IFNG;OAS2;IRF1;HLA-B;HLA-DPB1;HLA-DRA;HLA-F;GBP1; HLA-DQA2;* *HLA-DPA1*
GO:0042108	Positive regulation of cytokine biosynthetic process	3.37E−07	*SPN;CD4;CD80;IL1B;EBI3;TLR8;LTB;TNF*
GO:0002690	Positive regulation of leukocyte chemotaxis	4.35E−07	*CXCL10;CXCL9;CXCL11;CCL4;XCL2;CCL3;XCL1;FPR2;CXCL13*
GO:0001819	Positive regulation of cytokine production	7.41E−07	*GBP5;LILRB2;TNF;LY9;IFNG;CD6;IRF1;IL1B; HLA-DPB1;XCL1;CCL19;* *TIGIT;IL12RB1;HLA-DPA1*
GO:0071222	Cellular response to lipopolysaccharide	8.30E−07	*CXCL10;CD6;CD80;IL1B;CCL3;ACOD1;LILRB1;LILRB2;CCR5;TNF*
GO:0050870	Positive regulation of T cell activation	9.66E−07	*PTPRC;CD6;CD80;IL1B;HLA-DPB1;CCL19;LILRB2;CD3E; HLA-DPA1*

Only GO terms associated with biological processes with adjusted p value < 0.0001 are shown.

**Table 2:** Eighteen transcripts with high discriminative role between ISO-T and I + T (AUC = 1).

Transcript ID	Gene symbol	Official gene name	FC	FDR
ENST00000642037.1	**OR2I1P**	Olfactory receptor family 2 subfamily I member 1 pseudogene	12.70	7.93E−06
ENST00000641137.1	OR2I1P		11.71	1.14E−05
ENST00000641730.1	OR2I1P		10.48	1.90E−05
ENST00000452965.1	OR2I1P		14.56	3.07E−05
ENST00000444590.1	OR2I1P		12.81	3.57E−05
ENST00000447604.1	OR2I1P		14.76	9.25E−05
ENST00000450433.1	OR2I1P		13.66	9.25E−05
ENST00000453522.1	OR2I1P		11.24	0.0001
ENST00000428598.1	OR2I1P		14.59	0.000117
ENST00000370459.7	**GBP5**	Guanylate binding protein 5	15.66	1.14E−05
ENST00000429935.2	**UBD**	Ubiquitin D	11.09	2.23E−05
ENST00000377050.4	UBD		10.62	3.07E−05
ENST00000383547.3	UBD		10.25	0.000144
ENST00000522495.5	**IDO1**	Indoleamine 2,3-dioxygenase 1	21.23	5.54E−05
ENST00000479889.1	**GBP1**	Guanylate binding protein 1	8.15	6.52E−05
ENST00000370473.4	GBP1		7.70	9.74E−05
ENST00000443392.1	**CXCR2P1**	C-X-C motif chemokine receptor 2 pseudogene 1	24.75	6.67E−05
ENST00000306602.2	**CXCL10**	C-X-C motif chemokine ligand 10	14.94	6.67E−05

Cell type analysis performed in the Enrichr database using the Human Gene Atlas showed enrichment of NK cells (p = 1.6 *10^–8^), CD8 T cells (p = 8.5 *10^–8^), CD4 T cells (p = 3.5 *10^–4^), dendritic cells (p 5.2 *10^–4^), B cells (p = 0.0044) and monocytes (p = 0.028) in I + T biopsies compared to ISO-T.

### Interpretation of ISO-T and I + T by MMDx

**Figure 2:**
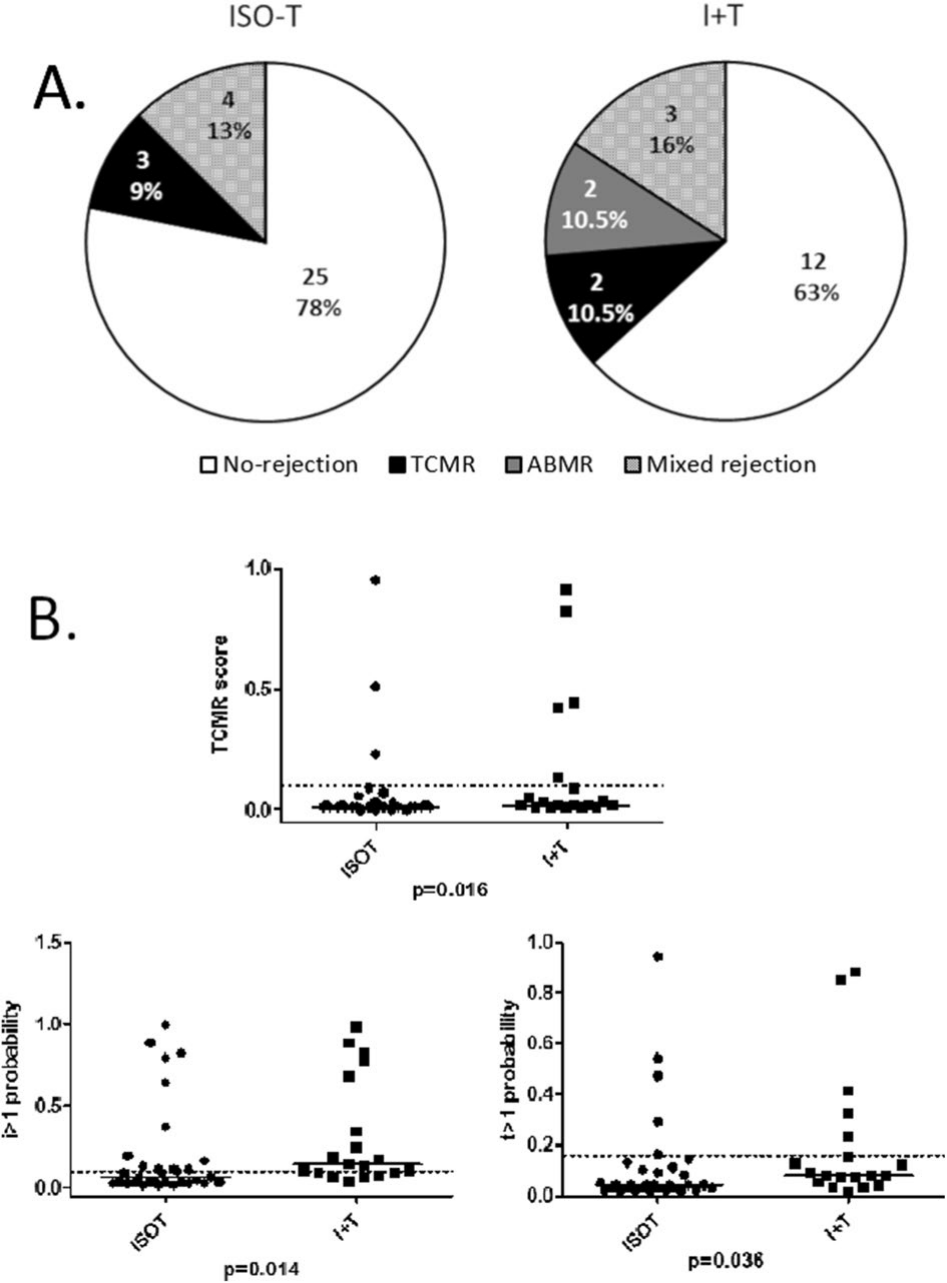
MMDx evaluation of the molecular phenotype in early indication biopsies with ISO-T (n = 32) and I + T (n = 19) categories of borderline changes. (**A**) Frequency of particular MMDx rejection diagnoses; (**B**) MMDx TCMR score and probability of moderate/severe tubulitis (t > 1) or interstitial inflammation (i > 1) scores.

To obtain the molecular diagnosis of kidney biopsies in this study, we used the well-established MMDx approach and compared rejection molecular scores, injury scores and the MMDx probability of histological scores in early indication biopsies (median 10 days post-transplantation, range 4–50 days) with ISO-T (n = 32) and compared them to I + T (n = 19) (Table S2). MMDx classified 25/32 cases of ISO-T as non-rejection (78%), 3/32 as T-cell mediated rejection (TCMR) (9%), and 4/32 as antibody-mediated rejection (ABMR) (13%). In the I + T group, MMDx classified 12/19 samples as no rejection (63%), 2/19 cases as TCMR (10.5%), 2/19 as ABMR (10.5%) and 3/19 as mixed rejection (16%) (Fig. 2A).

Several MMDx scores were lower in the ISO-T cohort: the TCMR score (p = 0.016), the t > 1 classifier (p = 0.036) and the i > 1 classifier (p = 0.014, Fig. 2B). Biopsies from the ISO-T category by histology also displayed weaker MMDx inflammatory signals. Of note, some of the ISO-T biopsies were called rejection by MMDx: two cases of severe TCMR, one case of mild TCMR, and 4 cases of mild ABMR. In cases of severe molecular TCMR, in protocol biopsies taken at 3 months post-transplant, classical histology again showed isolated tubulitis (i0t2) in the first case and chronic TCMR grade II in the second case. In one case, the early biopsy called mild TCMR by MMDx displayed mild glomerulitis (g1) in the 3 month protocol biopsy. In 1/4 mild ABMR cases called by MMDx in early biopsies, the protocol biopsy was called TCMR IIA, though concurrent SV40 positivity (polyomavirus) was also present.

### Overlap between transcripts increased in I + T compared to ISO-T revealed by RNAseq and pathogenesis related transcripts (PBTs)

Comparison of 461 transcripts increased in the I + T subcategory with previously published lists of PBTs showed significant enrichment of TCMR rejection-associated transcripts (TCMR-RATs)^14^ (14/30, 47%), QCATs^15^ representing cytotoxic T cell transcripts (11/24, 44%) and effector T cell transcripts^16^ (2/5, 40%), and ~ 10% enrichment of ABMR rejection-associated transcripts (ABMR-RATs)^17^ (4/30) and B-cell associated transcripts^18^ (6/100) (Table 3). This observation suggests that majority of I + T related transcripts are of T cell origin.

**Table 3:** Overlap between 461 increased transcripts in I + T compared to ISO-T revealed by RNAseq and pathogenesis related transcripts (PBTs).

PBTs/Classifier	Number of increased transcripts in I + T/number of transcripts in particular classifier (PBTs)	%	
TCMR classifier	14/30	46.7%	IFNG; IL12RB1; CD72; TIGIT; SH2D1A; CXCL13; ANKRD22; LAG3; CD8A; SLAMF8; ADAMDEC1; OR2I1P; IL21R; PLA2G2D
ABMR classifier	4/30	13.3%	CXCL10; CXCL11; CCL4; GNLY
**PBTs**			
ENDAT	0/119	0%	
GRIT	4/30	13.3%	HLA-DRA; UBD; CXCL9; PSMB9
QCAT	11/25	44%	IFNG; CD3D; GZMB; CD8A; GZMA; CST7; CD2; GZMK; CXCR6; GNLY; NKG7
QCMAT	7/67	10.4%	LILRB4; LILRB2; TLR8; LYZ; SLAMF8; ADAMDEC1; IL4I1
DSAST	1/21	4.8%	GNLY
AMAT	1/10	10%	MMP9
IRRAT	1/30	3.3%	PTPRC
TCB	2/5	40%	CD3D; CXCR6
BAT	6/100	6%	GABBR1; SPIB; CD72; PAX5; CD79A; LY9
MCAT	1/4	25%	TPSB2

ENDAT, endothelial cell associated transcripts; GRIT, IFN-gamma and rejection induced transcripts; QCAT, quantitative CTL-associated transcripts; QCMAT, quantitative constitutive macrophage-associated transcripts; DSA, donor-specific antibody (DSA) selective transcripts; AMAT; alternative macrophage associated transcripts; IRRAT, injury-repair response associated transcripts; TCB, T cell transcript burden; BAT, B-cell associated transcripts; MCAT, mast cell associated transcripts.

## Discussion

In this study we analysed the molecular phenotypes of histologic isolated tubulitis and inflammation with tubulitis, which formerly made up the borderline (suspicious) for acute TCMR diagnostic category^5,6^. The threshold for the interstitial inflammation score (i) has not been used uniformly by different pathologists^19^. Therefore, based on a recent study of Nankivell et al.^7^ showing no effect of isolated t-lesions on graft outcome, the Banff 2019 conference set the minimum lesion requirement for borderline diagnosis as i1t1. In our study, molecular profiling by both RNAseq and MMDx clearly distinguished between ISO-T and I + T subcategories, revealing less activation of inflammatory processes in ISO-T.

In our study, RNA sequencing of biopsies with histologic interstitial inflammation showed transcripts associated with activation of cytokine-mediated signalling pathways, response to interferon-gamma, inflammatory response and other GO terms associated with lymphocyte chemotaxis and cytokine production. Cell type analysis of increased transcripts in biopsies with interstitial inflammation (I + T) showed significant enrichment of NK cells, CD8 and CD4 T cells, followed by dendritic cells, B cells and monocytes suggesting the involvement of both innate and adaptive immune cells. It is known that macrophages are a major component of interstitial infiltrates in renal allografts^20^. Neither cell type analysis or analysis of 67 individual macrophage-associated transcripts^21^ found significant enrichment of macrophage-associated transcripts in early indication biopsies with interstitial inflammation compared to ISO-T. One reason why we did not find macrophage enrichment may be that markers for this cell population are not clearly defined. Recently, *CXCL11* and *CCL19* were found to be highly expressed in inflammatory macrophages compared with other cell types^22^ and distinguished acute rejection from stable patients. This corresponds with the results of our study, where both *CXCL11* and *CCL19* were highly increased in I + T vs. ISO-T (*CXCL11* fold change = 17, adjusted p value = 0.00025; *CCL19* fold change = 13, adjusted p value = 0.0019).

The less inflammatory phenotype of ISO-T as revealed by RNAseq was confirmed by MMDx with lower TCMR, interstitial inflammation and tubulitis molecular scores. Of note, 22% of ISO-T and 38% of I + T biopsies were still classified as rejection by MMDx. The evaluation of biopsies with minimal injury by MMDx system may reveal which biopsies are more likely molecular rejections, however this system has not yet been widely implemented in the clinical praxis, due to several obstacles including its high costs and until now centralized global laboratory. Similarly, 1/8 samples in ISO-T and 7/8 samples in I + T displayed significant inflammatory signals by RNA sequencing. This illustrates the issue of attempting to dichotomize data instead of approaching the interpretation probabilistically. It remains unclear whether this particular molecular rejection phenotype is predictive of worse graft survival when all patients had received steroid pulses and mid-term outcome was similar.

In summary, isolated tubulitis within the first 2 weeks post-transplant diagnosed by histology displayed fewer inflammatory signals by molecular assessment compared to tubulitis with interstitial inflammation—supporting the idea that this is a benign phenotype.

## Materials and methods

### Study design

Evaluation of kidney graft outcome in ISO-T and I + T categories was performed on cohort of patients where those categories were identified in early indication biopsies (performed at median 9 post-operative days) as a solely and first pathology. All kidney pathology records were retrospectively reviewed to identify biopsies with ISO-T and I + T in early indication biopsies (n = 338) performed between January 2005 and January 2017. Cases with surgical complications, previous or concurrent rejections, thrombotic microangiopathy (TMA), glomerulonephritis recurrence, glomerulitis > 1, BKV nephropathy and those who received no steroid pulses to cure BL were excluded (Fig. 1). The final study cohort consisted of 126 I + T and 135 ISO-T biopsies.

**Figure 3:**
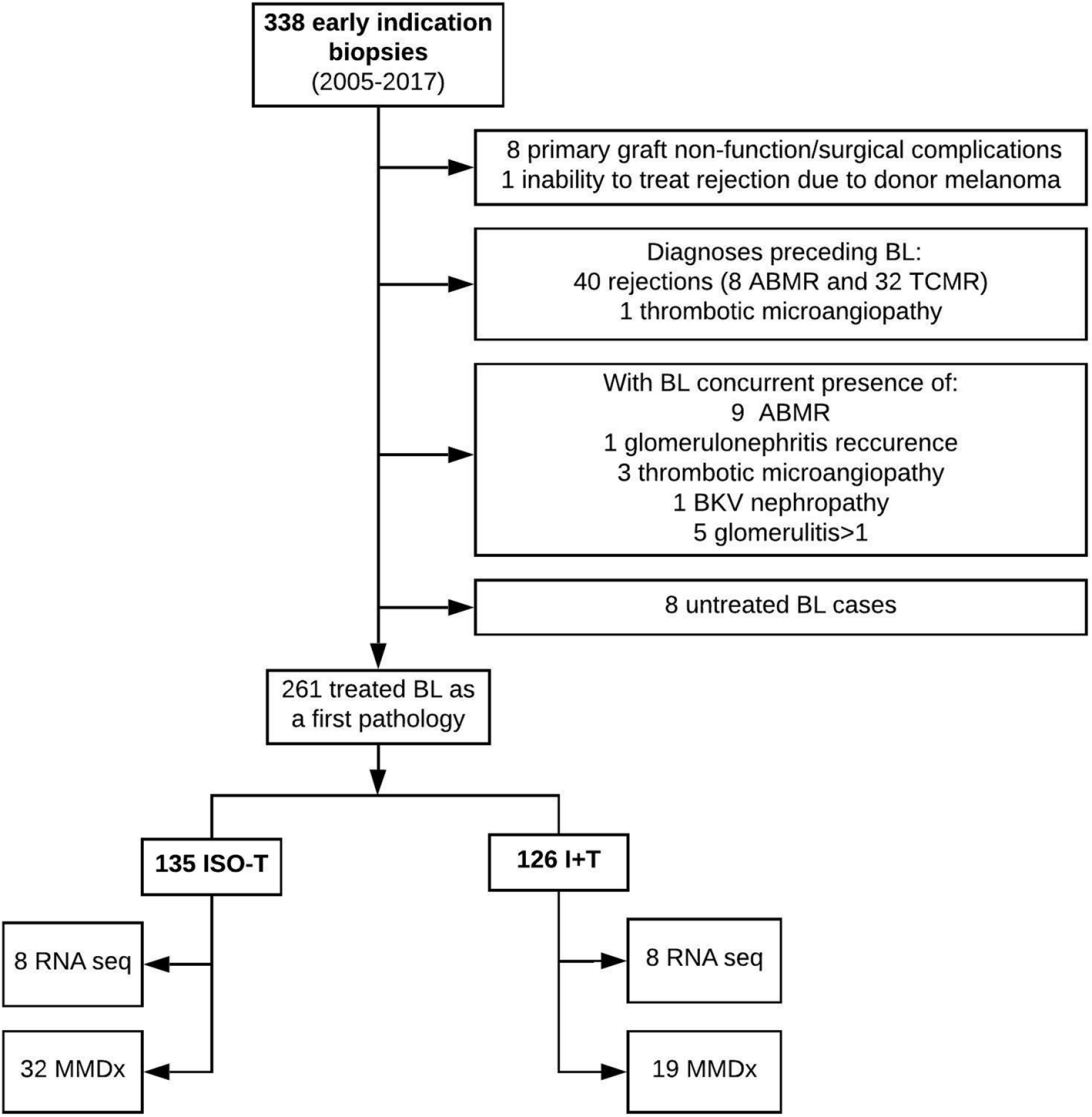
Flow chart of study participants’ enrolment.

Molecular phenotypes of both ISO-T and I + T histological findings were studied using either RNA sequencing or MMDx in two different sub-cohorts with available biopsy samples stored in the biobank for transcriptomic analysis (Fig. 3). RNA sequencing analysis was studied in 8 ISO-T and 8 I + T biopsies. Patient demographics are given in Supplemental Table S1. MMDx diagnostics were performed in 32 ISO-T and 19 I + T biopsies. Both groups of patients had similar transplant demographics (Supplemental Table S2).

This study was approved by the local Ethics Committee of the Institute for Clinical and Experimental Medicine and Thomayer Hospital under No. G-16–06-09, and IKEM biobanking was approved under A 13–02-01 (83/13). All methods were carried out in accordance with relevant guidelines and regulations. All patients provided written informed consent, and the study was conducted according to the principles of the Declaration of Helsinki and Istanbul.

### Histopathology and definition of BL

Kidney allograft biopsy samples were obtained using a percutaneous ultrasound-guided 16G biopsy needle. The ISO-T category was defined as isolated tubulitis without interstitial inflammation (Banff i0t1, i0t2, i0t3) and I + T category as mild interstitial inflammation with tubulitis (Banff i1t1, i1t2, i1t3) or mild tubulitis with moderate/severe interstitial inflammation (Banff i2t1, i3t1). A small piece of the biopsy specimen (2–4 mm) cut from the middle of biopsy core was immediately placed in RNA*later™* (Qiagen) and stored at − 80 °C in the biobank for transcriptomic analysis.

### RNA sequencing

To perform RNA sequencing in 8 ISO-T and 8 I + T biopsies, total RNA was isolated from biopsy specimens stored at -80 °C in RNA*later™* (Qiagen) using the RNeasy Micro Kit (Qiagen, Hilden, Germany). RNA concentration was measured by Qubit fluorimeter and RNA integrity number was checked using Agilent Bioanalyzer 2100. From 400 ng of total RNA, mRNA was isolated using poly (A) magnetic selection NEBNext® Poly (A) mRNA magnetic isolation module (New England, BioLabs, Inc). Transcriptome libraries for differential gene expression were prepared using the NEBNext® Ultra™ II Directional RNA Library Prep with Sample Purification Beads according to the manufacturer’s protocol (New England, BioLabs, Inc). In brief, mRNA was randomly sheared by heat digestion in the presence of a divalent metal cation (Mg^2+^). Sheared RNA was reversibly transcribed making 1^st^ strand of cDNA using random hexamers as primers and reverse transcriptase. The second strand was created using dUTPs, purified with Sample Purification Beads, and ligated with NEBNext adapters. After removal of the 2^nd^ strand by uracil-DNA-dependent glycosylase, the final amplification of adaptor-ligated DNA was done using NEBNext® Multiplex Oligos for Illumina®. Library quality was assessed on a Bioanalyzer 2100 using the Agilent High Sensitivity DNA 1000 assay. Libraries from all 16 samples were pooled to a final concentration of 50 nmol and the quality of pooling was assessed by sequencing using MiSeq. High throughput sequencing of the final pool was performed using NovaSeq6000 S4 system (Illumina) with following instrument settings: single-end, 100 b, 300–400 million reads per lane. In total, 1,074,246,287 single-end 100 b reads were generated.

Raw data were automatically processed by Basespace cloud interface (Illumina) in default settings. The basecalling, adapter clipping, and quality filtering were carried out using bcl2fastq v2.20.0.422 Conversion Software (Illumina).

The quality of raw reads was evaluated using FastQC (v0.11.8) and MultiQC (v1.7). Clipping adaptor sequences was carried out using cutadapt (v1.18). The trimmed reads were aligned to the human transcriptome reference (GRCh38) using bowtie2 (v2.3.4.3). The alignments were evaluated using qualimap2 (v2.2.2). The counts of reads mapped to the reference were extracted and used for differential gene expression analysis using SAMtools (v1.9). The differential gene expression analysis was performed using DESEq2 and EdgeR packages in R (v3.4.4). The transcripts with log fold change > 2 or < -2 and with p-value less than 0.05 were considered as significantly differentially expressed. Overlap of differentially expressed transcripts determined by both methods were considered as significant. Gene annotation analysis was performed using Enrichr (https://amp.pharm.mssm.edu/Enrichr/)^23,24^. Complete raw and normalized data were deposited in the NCBI Gene Expression Omnibus (GEO) database^25^ and can be accessed using the GEO Series accession number GSE161705.

### Molecular microscope diagnostic system analysis

Biopsy specimens in RNA*later™* (Qiagen) were sent on dry ice to the Alberta Transplant Applied Genomics Centre (ATAGC, University of Alberta) for MMDx analysis. RNA extraction and gene expression analysis using PrimeView GeneChip arrays (Affymetrix, Santa Clara, CA) were performed as previously described^9^. Classifiers related to rejection (ABMR, TCMR, and all rejection) or acute kidney injury (AKI), inflammation and chronic injury (atrophy/fibrosis score) were generated using a recently published reference set of 1208 biopsy specimens^11^.

### Statistical analysis

Continuous variables were expressed as medians and min/max values or by means and standard deviations. Categorical variables were expressed as the frequency and the percentage of the total. As most data sets failed to exhibit standard normal distribution (based on the Kolmogorov–Smirnov test), non-parametric statistical methods were used for further analysis. Data sets were compared using the Mann–Whitney U test with categorical data compared using Fisher’s exact test. P values < 0.05 were considered statistically significant. Data analysis was performed using IBM SPSS Statistics 24 and GraphPad Prism5 software.

## Supplementary information

Supplementary Information 1.
