# Author Correction: Molecular patterns of isolated tubulitis differ from tubulitis with interstitial inflammation in early indication biopsies of kidney allografts

**DOI:** 10.1038/s41598-021-88190-y

**Published:** 2021-04-14

**Authors:** Petra Hruba, Katelynn Madill-Thomsen, Martina Mackova, Jiri Klema, Jana Maluskova, Ludek Voska, Alena Parikova, Janka Slatinska, Philip F. Halloran, Ondrej Viklicky

**Affiliations:** 1 Transplant Laboratory, Institute for Clinical and Experimental Medicine, 140 21 Prague, Czech Republic; 2 Alberta Transplant Applied Genomics Centre, Edmonton, AB T6G 2S2 Canada; 3 University of Alberta, Edmonton, AB T6G 2S2 Canada; 4 Department of Computer Science, Faculty of Electrical Engineering, Czech Technical University, 121 35 Prague, Czech Republic; 5 Department of Pathology, Institute for Clinical and Experimental Medicine, 140 21 Prague, Czech Republic; 6 Department of Nephrology, Transplant Centre, Institute for Clinical and Experimental Medicine, Videnska 1958/9, 14021 Prague, Czech Republic

Correction to: *Scientific Reports* 10.1038/s41598-020-79332-9, published online 17 December 2020

The original version of this Article contained a typographical error in the spelling of the author Katelynn Madill-Thomsen, which was incorrectly given as Katellyn Madill-Thomsen.

This error has now been corrected in the PDF and HTML versions of the Article, and in the accompanying Supplementary Information file.

