# Supplementary material for: Molecular patterns of isolated tubulitis differ from tubulitis with interstitial inflammation in early indication biopsies of kidney allografts
Source: Sci Rep. 2020 Dec 17;10:22220. doi: 10.1038/s41598-020-79332-9 (PMC7746707; doi:10.1038/s41598-020-79332-9)
Supplement: Supplementary file 1 — Supplementary Information 1. [file 41598_2020_79332_MOESM1_ESM.doc]

**Molecular patterns of isolated tubulitis differ from tubulitis with interstitial inflammation in early indication biopsies of kidney allografts**

Petra Hruba ^1^, Katelynn Madill-Thomsen ^2,3^, Martina Mackova ^2^, Jiri Klema^4^, Jana Maluskova ^5^, Ludek Voska ^5^, Alena Parikova ^6^, Janka Slatinska ^6^, Philip F Halloran ^2^, and Ondrej Viklicky ^1,4^

**Supplementary Material**

**Table S1** Characteristic of patients analysed by RNAseq.

|  | **ISO-T (n=8)** | **I+T (n=8)** | P value |
| --- | --- | --- | --- |
| Time of biopsy (days after Tx) | 9 [6; 18] | 7 [5; 15] | 0.339 |
| Creatinine at biopsy (µmol/L) | 371 [290;926] | 335 [145;723] | 0.345 |
| Type of donor, deceased, n (%) | 8 (100%) | 6 (75%) | 0.301 |
| Donor age, years | 55 [38;67] | 51 [19;62] | 0.400 |
| HLA mismatch | 4 [3;6] | 4 [2;6] | 0.956 |
| Peak PRA | 3 [0;25] | 2.5 [0;89] | 0.669 |
| Cold ischemia, hours | 19 [14;24] | 16 [1;18] | 0.155 |
| T cell depletive induction | 0 | 3 (37.5%) | 0.117 |

**Table S2** Characteristic of patients analysed by MMDx.

|  | **ISO-T (n=32)** | **I+T (n=19)** | **p value** |
| --- | --- | --- | --- |
| Time of biopsy (days after Tx) | 11 [4;50] | 10 [5;50] | 0.754 |
| Creatinine at biopsy (µmol/L) | 348 [119;820] | 221 [99;767] | 0.199 |
| Type of donor, deceased, n (%) | 23 (72%) | 15 (79%) | 0.743 |
| Donor age, years | 53 [24;80] | 50 [29;74] | 0.807 |
| HLA mismatch | 3 [1;5] | 3 [1;5] | 0.497 |
| Peak PRA | 3 [0;73] | 6 [0;46] | 0.336 |
| Cold ischemia, hours | 15 [0;24] | 15 [0;24] | 0.302 |
| T cell depletive induction | 6 (18.8%) | 4 (21%) | 0.169 |

**Figure S1** A. Graft survival and B. acute rejection-free interval (subclinical rejections found in protocol biopsies of patients with stable graft function were excluded) in ISO-T and I+T cohorts analysed by MMDx (n=51) and RNAseq (n=16). C. acute rejection-free interval in patients diagnosed in the first biopsy by MMDx as rejection positive (MMDx+) or negative (MMDx-). Death-censored survival analyses were calculated using Log-rank test.
